# Oct4-mediated reprogramming induces embryonic-like microRNA expression signatures in human fibroblasts

**DOI:** 10.1038/s41598-019-52294-3

**Published:** 2019-10-31

**Authors:** Lucie Peskova, Katerina Cerna, Jan Oppelt, Marek Mraz, Tomas Barta

**Affiliations:** 1 Department of Histology and Embryology, Faculty of Medicine, Masaryk University, Brno, 625 00 Czech Republic; 2 CEITEC-Central European Institute of Technology, Masaryk University, Brno, 625 00 Czech Republic; 3 Department of Internal Medicine, Hematology and Oncology, University Hospital Brno and Faculty of Medicine, Masaryk University, Brno, Czech Republic; 4 National Centre for Biomolecular Research, Faculty of Science, Masaryk University, Brno, 625 00 Czech Republic

**Keywords:** Stem-cell biotechnology, miRNAs, Reprogramming

## Abstract

Oct4-mediated reprogramming has recently become a novel tool for the generation of various cell types from differentiated somatic cells. Although molecular mechanisms underlying this process are unknown, it is well documented that cells over-expressing Oct4 undergo transition from differentiated state into plastic state. This transition is associated with the acquisition of stem cells properties leading to epigenetically “open” state that is permissive to cell fate switch upon external stimuli. In order to contribute to our understanding of molecular mechanisms driving this process, we characterised human fibroblasts over-expressing Oct4 and performed comprehensive small-RNAseq analysis. Our analyses revealed new interesting aspects of Oct4-mediated cell plasticity induction. Cells over-expressing Oct4 lose their cell identity demonstrated by down-regulation of fibroblast-specific genes and up-regulation of epithelial genes. Interestingly, this process is associated with microRNA expression profile that is similar to microRNA profiles typically found in pluripotent stem cells. We also provide extensive network of microRNA families and clusters allowing us to precisely determine the miRNAome associated with the acquisition of Oct4-induced transient plastic state. Our data expands current knowledge of microRNA and their implications in cell fate alterations and contributing to understanding molecular mechanisms underlying it.

## Introduction

The forced expression of defined transcription factors in cells is capable of inducing dramatic cell-fate conversions. The combination of four transcription factors Oct4, Sox2, Klf4, and c-Myc (abbreviated as OSKM) induces pluripotency reprogramming in somatic cells leading to the generation of induced pluripotent stem cells (iPSCs) with the capacity to differentiate into multiple specific cell types^1,2^. Other transcription factors have been shown to directly convert differentiated somatic cell types, a process called direct cell lineage conversion or transdifferentiation while bypassing the pluripotent state (reviewed in^3^).

Recently, an alternative reprogramming approach has been introduced. It involves a brief exposure of differentiated somatic cells to pluripotency-associated transcription factors, leading to de-differentiation and acquisition of a stem cell-like, epigenetically “open/plastic” state that is permissive to external stimuli towards differentiation, potentially without full reversion to pluripotent state^4–7^. However, increasing number of studies suggests that this approach leads to a transient induction of pluripotency^8,9^. Still, this method has the advantage of higher efficiency, elevated proliferation, and erasure of epigenetic memory, when compared to direct cell lineage conversion^7^. Furthermore, this approach generates rejuvenated multipotent progenitor cell populations that are able to differentiate into specific cell types, hence lacking all limitations typical for transdifferentiated cells. To date, cells of neuronal, cardiac, hepatocyte, endothelial or smooth muscle fate have been generated through the cell plasticity induction^10–13^.

Cell plasticity induction is commonly driven by short ectopic expression of factors typically used for reprogramming to iPSCs^14,15^. Among these, Oct4 has been shown to be the master regulator of reprogramming and cell plasticity induction^5,13,16,17^. Oct4 over-expression has been shown to generate hematopoietic and neural progenitors^4–6^ and in a combination with small molecules has proven to be sufficient to reprogram fibroblasts into iPSCs^18–20^.

The molecular mechanisms underlying Oct4-mediated reprogramming are not fully understood. It has been demonstrated that Oct4 expression induces state unlike that of being observed during reprogramming to iPSCs with a lack of molecular hallmarks of iPSCs formation^4,5^. This plastic state is characterised by loss of cell identity, de-differentiation and onset of a stem cell-like state associated with mixed expression of developmentally related genes that are linked to multiple lineages, but not with pluripotency status^5^.

Even though it is well established that transcription factor-driven cell reprogramming requires extensive changes in chromatin structure, epigenetic modifications, signalling pathways and molecular profiles, the precise molecular signature and mechanisms of Oct4-induced plasticity remain elusive. The short non-coding RNAs – namely microRNAs (miRNAs) – have emerged as important regulators of cell pluripotency and reprogramming^21^. MiRNAs regulate virtually all cellular processes including cell fate specification by targeting 3´UTR of messenger RNAs (mRNAs), which leads to translational repression or mRNA degradation^22,23^. There is a lot of information on the expression of miRNAs during OSKM over-expression and forced induction or repression of specific miRNAs is known to positively impact reprogramming to pluripotency^21,24,25^, or even induce pluripotent state in somatic cells^26–29^. Despite the fact that Oct4-induced cell plasticity has the same de-differentiation outcome as OSKM over-expression, it is not clear how miRNAs are involved in this process. Therefore, we aimed to characterise cells ectopically expressing Oct4 and to identify miRNA networks involved in Oct4-mediated cell plasticity. We found that human dermal fibroblasts (hDFs) over-expressing Oct4 lose their cell identity demonstrated by down-regulation of fibroblast-specific genes and elevated expression of epithelial genes. We revealed a differential miRNA expression profile in hDFs versus hDFs over-expressing Oct4. Surprisingly, our data shows an embryonic-like miRNA expression pattern as soon as 6 days upon Oct4 over-expression in hDFs that is rather typically found at a later stage of reprogramming process or in pluripotent stem cells^24^. Altogether, our data indicates that Oct4-mediated reprogramming may operate through unique molecular mechanisms associated with different kinetics, when compared to classical OSKM over-expression approach. This study provides a novel insight into understanding of the molecular profile of cell plasticity, which is necessary for reprogramming strategies optimisations and their successful implementations.

## Results

### Oct4 over-expression down-regulates mesenchymal and fibroblast-specific genes and up-regulates epithelial genes

We transduced human dermal fibroblasts (hDFs) with lentiviral vectors for Oct4 over-expression and GFP for control experiments. We chose hDFs, because they are readily obtainable from patient skin biopsies, easily cultured *in vitro* and typically used as a starting cell line in reprogramming experiments. To determine the nature of Oct4-induced plasticity, hDFs were cultured in hDFs media in the absence of lineage-inducing growth factors that have been used in previous studies^4–6^, as the presence of these factors would bring bias to our analysis. Cells were harvested 6 days upon transduction and Puromycin selection. We chose this time-point, because 6 days provides enough time for antibiotic selection to yield homogenous population of cells expressing Oct4.

**Figure 1:**
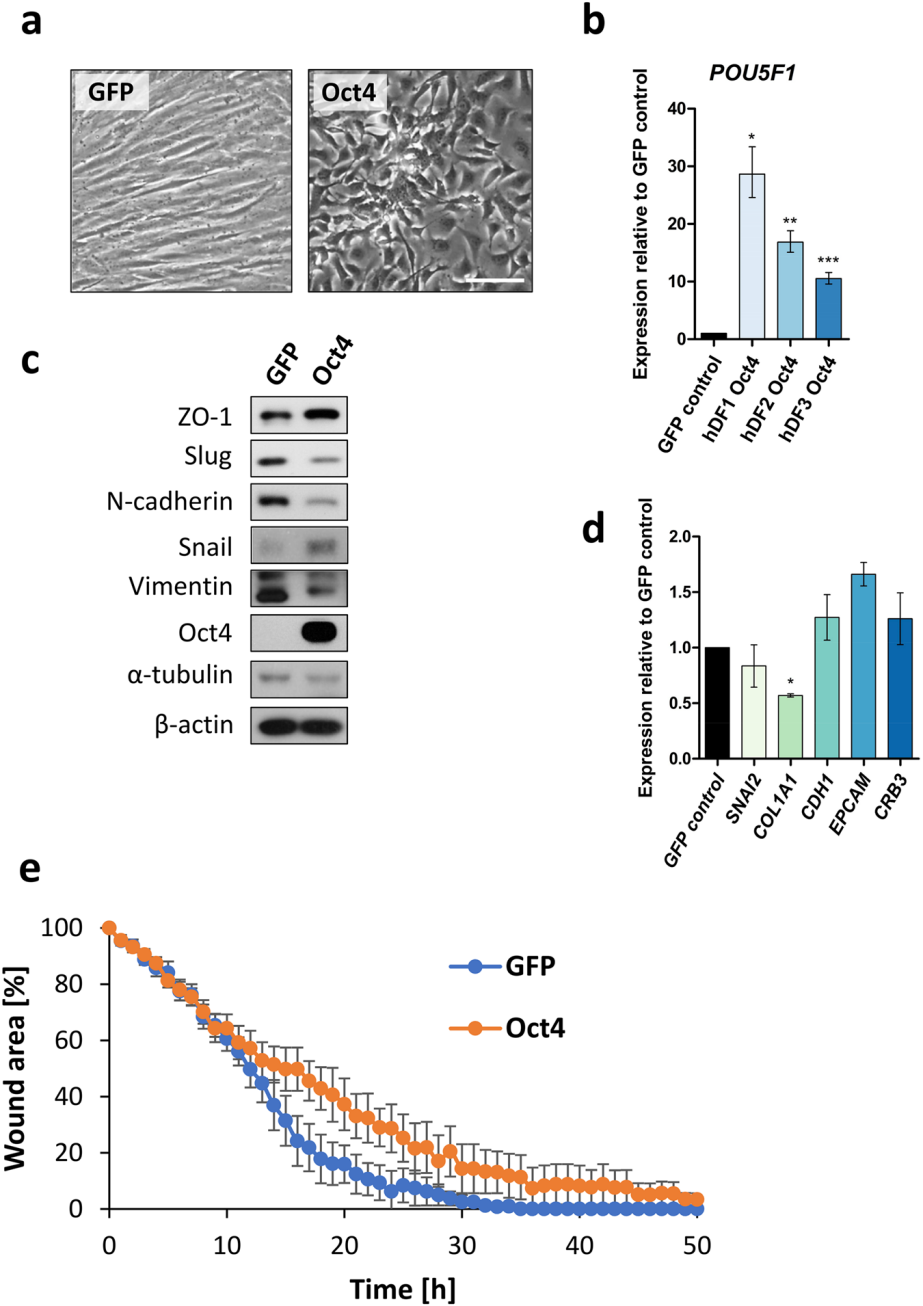
Characterisation of Oct4+ hDFs. **(a)** Morphology of control GFP+ hDFs and Oct4+ hDFs 6 days post transduction, as determined by light microscopy. Scale bar = 100 μm. **(b)** Analysis of *POU5F1* expression in Oct4+ hFDs relative to GFP+ hDFs 6 days post transduction, as determined by RT-qPCR. Error bars represent ± SD. **(c)** Western blot analysis of mesenchymal/epithelial markers and Oct4 expression in control GFP+ hDFs and Oct4+ hDFs 6 days post transduction. α-tubulin and β-actin were used as a loading control. Uncropped western blot images are shown in Supplementary Fig. 2a. **(d)** Analysis of *SNAI2, COL1A, CDH1, EPCAM, CRB3* expression in Oct4+ hFDs relative to GFP+ hDFs 6 days post transduction, as determined by RT-qPCR. Error bars represent ± SD. **(e)** Analysis of cell migration of Oct4+ and GFP+ hDFs, as determined by scratch-wound healing assay. The graph shows cell-free area during time upon making a straight scratch on tissue culture plate. Error bars show ± SE, n = 5.

Transduced hDFs over-expressed Oct4, showed a dramatic change of morphology shortly upon Oct4 over-expression with transition of long-spindled fibroblast morphology to short-spindled cell shape (Fig. 1a,b), and maintained this altered morphology for at least 30 days (Fig. S1). In order to further analyse molecular mechanisms underlying changed morphology, we aimed to assess the expression of mesenchymal and epithelial genes. Western blot analysis revealed down-regulation of mesenchymal and fibroblast markers such as Slug, N-cadherin, Vimentin and up-regulation of epithelial marker ZO-1 (Figs 1c, S2a). Interestingly, we also detected up-regulation of Snail (*SNAI1*), which is classically viewed as a mesenchymal gene, however, we and others have previously shown that Snail is up-regulated during early stages of reprogramming and enhances efficiency of iPSCs generation^25,30^. RT-qPCR analysis showed down-regulated mesenchymal genes *COL1A1, SNAI2* (*SNAI2* not significantly) and not significantly up-regulated epithelial genes *CDH1*, *EPCAM*, and *CRB3* upon Oct4 over-expression (Fig. 1d).

Given the observed change of cell morphology and altered mesenchymal/epithelial gene expression, we sought to further investigate, if Oct4 over-expression affects cell migration. Scratch-wound assay showed that control (GFP+) hDFs rapidly filled cell-free area in ~30 hours upon making a scratch, while Oct4+ hDFs were much slower filling cell-free area in ~50 hours (Figs 1e and S3), indicating that Oct4 over-expression impairs cell migration.

Altogether, the observed change of Oct4+ cells morphology, changes in the levels of mesenchymal- and epithelial-related markers, and slower cell migration might suggest that hDFs undergo mesenchymal-to-epithelial transition (MET) during Oct4-induced cell plastic state.

### miRNA-Seq results: sample to sample variation and quality check

MiRNA expression was analysed using three independent biological replicates represented by three different hDF cell lines expressing Oct4 or GFP respectively (here referred to as hDF1-3 Oct4 or hDF1-3 GFP).

**Figure 2:**
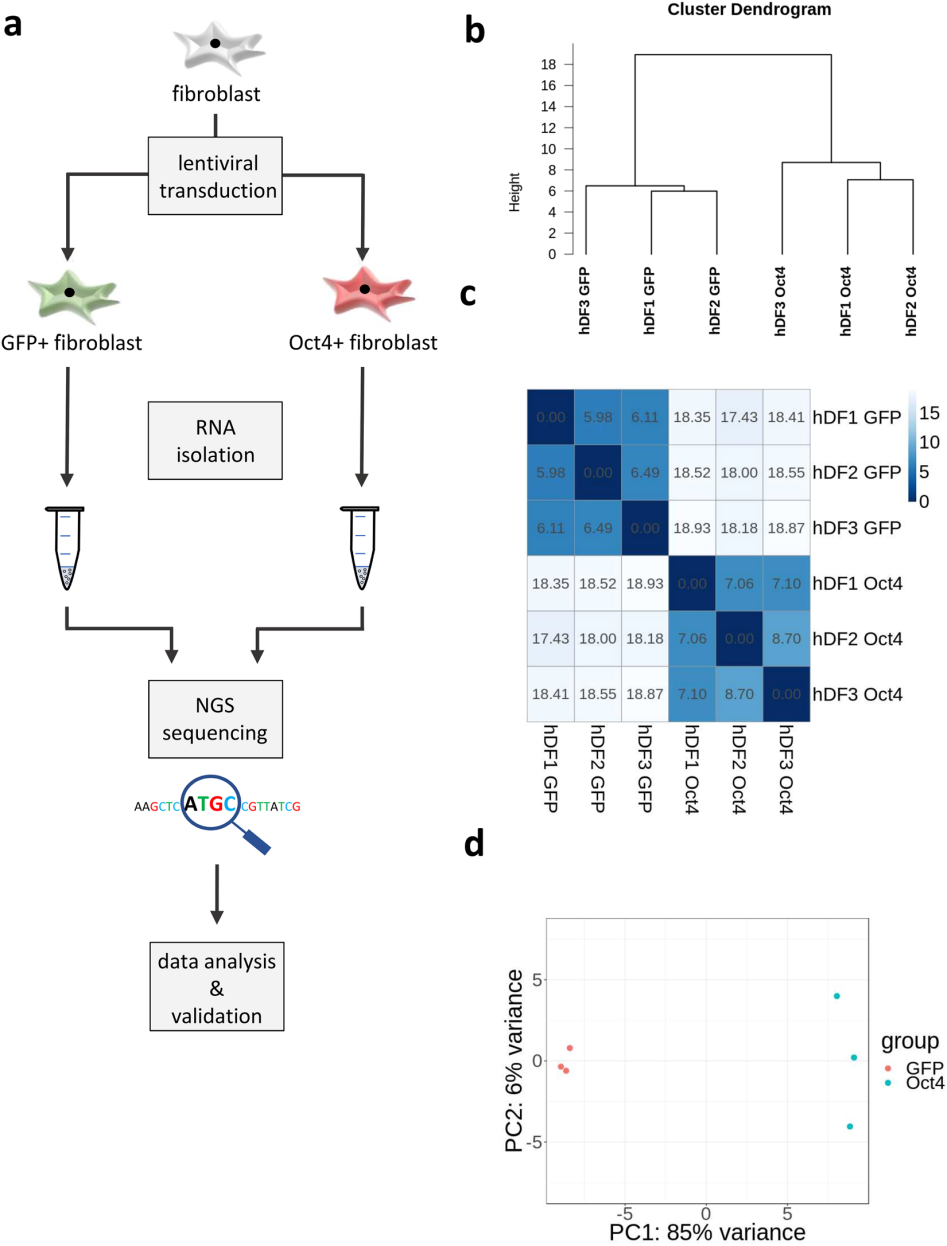
Variation of miRNA expression between Oct4+ and GFP+ hDFs. **(a)** Scheme illustrating experimental scenario. **(b)** Hierarchical clustering, **(c)** heatmap, and **(d)** PCA analysis showing differences in miRNA expression between Oct4+ and GFP+ hDFs in each replicate.

At day 6 post transduction and antibiotic selection, total RNA was isolated from control GFP+ and Oct4+ hDFs (see Fig. 2a for the experimental design) and subjected to miRNA-Seq. Every biological replicate contained more than 4.5 × 10^6^ non-filtered reads and Cook’s distance analysis did not reveal any outliers among sequenced biological samples (Fig. S5). Hierarchical clustering, PCA analysis, and correlation matrix between samples showed highly distinct miRNA expression profiles between Oct4+ and GFP+ hDF cells, while there was no significant intra-group variation from sample to sample (Fig. 2b–d).

### miRNA-Seq results: differentially expressed miRNAs

**Figure 3:**
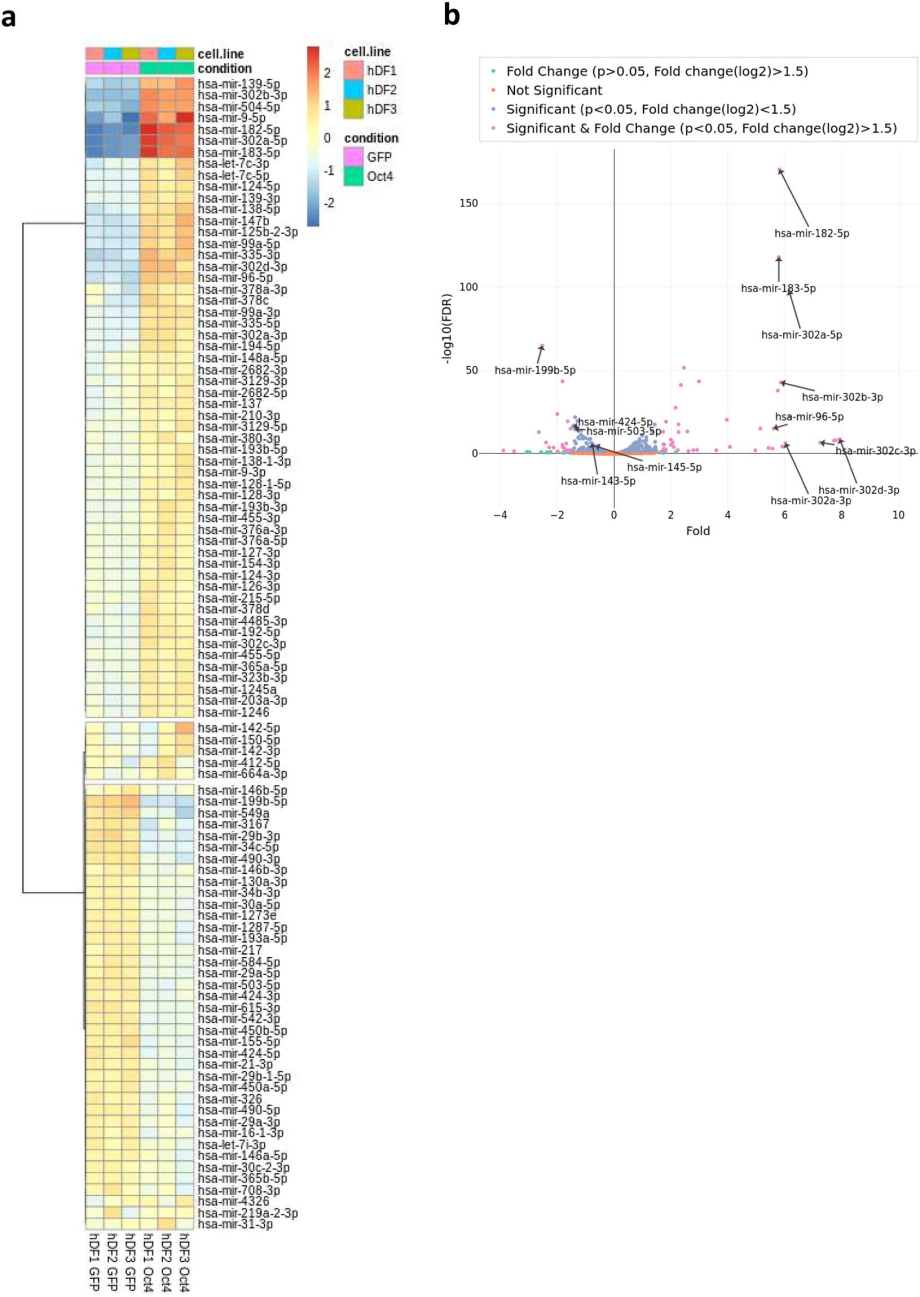
miRNA expression associated with Oct4-induced plastic state. **(a)** Heatmap shows the expression of miRNAs in three biological replicates of Oct4+ and GFP+ hDFs. **(b)** Pairwise analysis (volcano plot) of miRNAs expression between Oct4+ and GFP+ hDFs. Purple dots represent significantly (p < 0.05, Fold change(log2) > 1.5) differentially expressed miRNAs.

Given the striking difference in miRNA expression profile between Oct4+ and GFP+ hDFs, we sought to further characterize miRNAs in cell plasticity induction process. We detected 1,654 miRNAs from approximately 2,600 human mature miRNAs described in miRBase database^31^, while 212 were significantly (p < 0.05) differentially expressed between Oct4 and GFP expressing hDFs. When more stringent criteria (p < 0.05, log2(fold change) > 1.5) were applied, then 42 miRNAs were significantly up-regulated and 20 down-regulated in Oct4+ hDF when compared to GFP controls (Fig. 3a,b).

**Figure 4:**
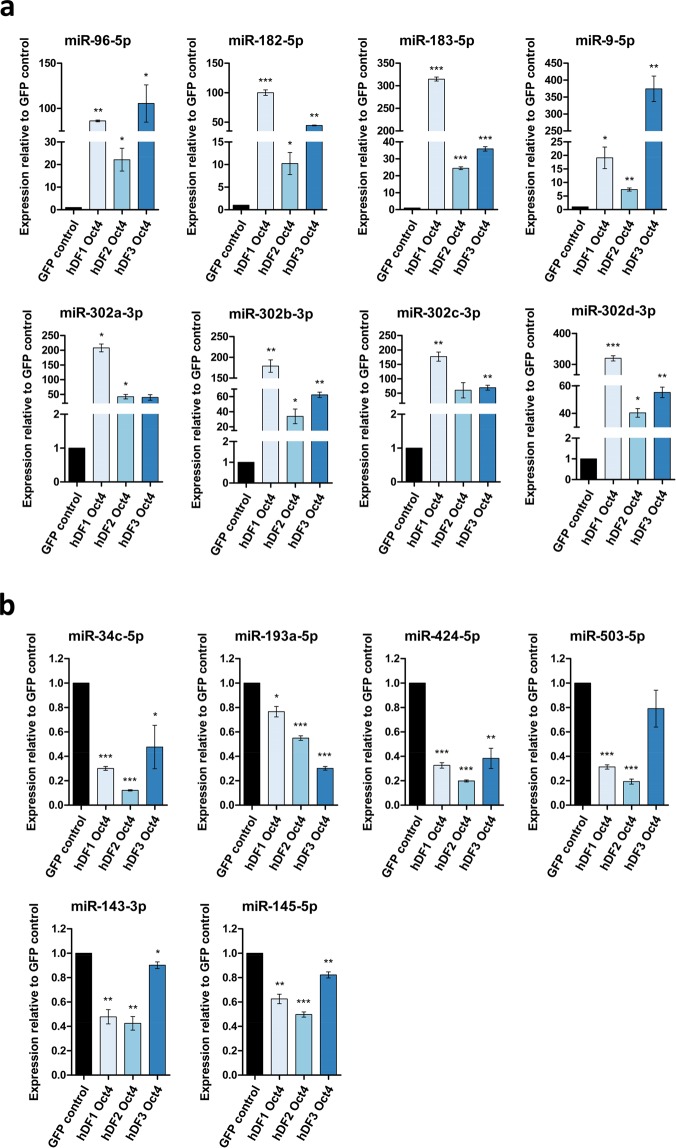
Validation of NGS using RT-qPCR. Expression levels of selected miRNAs that are differentially expressed based on NGS data, as determined by RT-qPCR. **(a)** miRNAs up-regulated in Oct4+ hDFs: mir-183, mir-302 cluster members, and miR-9-5p. **(b)** miRNAs down-regulated in Oct4+ hDFs: miR-34c-5p, miR-193a-5p, miR-424-5p, miR-503-5p, miR-143-3p and miR-145-5p. The results are shown as expression relative to corresponding control GFP+ hDFs. Error bars represent ± SD.

The expression of several differentially expressed miRNAs (up-regulated: miR-96-5p, miR-182-5p, miR-183-5p, miR-9-5p, miR-302a,b,c,d-3p; down-regulated: miR-34c-5p, miR-193a-5p, miR-424-5p, miR-503-5p, miR-143-3p, miR-145-5p) was confirmed using RT-qPCR demonstrating that the expression of selected miRNAs was in concordance with the miRNA-Seq data (Fig. 4a,b). It is of note that the expression of some miRNAs is different among the replicates. This may be caused by different levels of Oct4 expression among the replicates, as the expression of some miRNAs (e.g. members of mir-302 cluster) correlates with the expression level of Oct4 in individual hDF cell lines (Figs 1b and 4a). Additionally, triplicates represent three different cell lines derived from three different individuals, thus the variability of miRNA expression might be also contributed to the nature of distinct hDF cell lines.

To address the question, whether changes to the miRNAs expression upon Oct4 overexpression is temporal or permanent, we assessed the expression of key selected miRNAs at day 6 and 20 upon transduction (Fig. S6). Although the expression of Oct4 gradually increases, reaching ~900 fold change at day 20, not all miRNAs shared similar expression pattern, indicating that Oct4-induced plastic state is not stable at miRNA expression level. Some miRNAs from mir-302 cluster (miR-302a-3p, miR-302b-3p) were significantly up-regulated at day 20, while miR-193a-5p and miR-145-5p were significantly down-regulated, when compared to day 6.

### miRNA-Seq results: genomic clusters and family analyses

**Figure 5:**
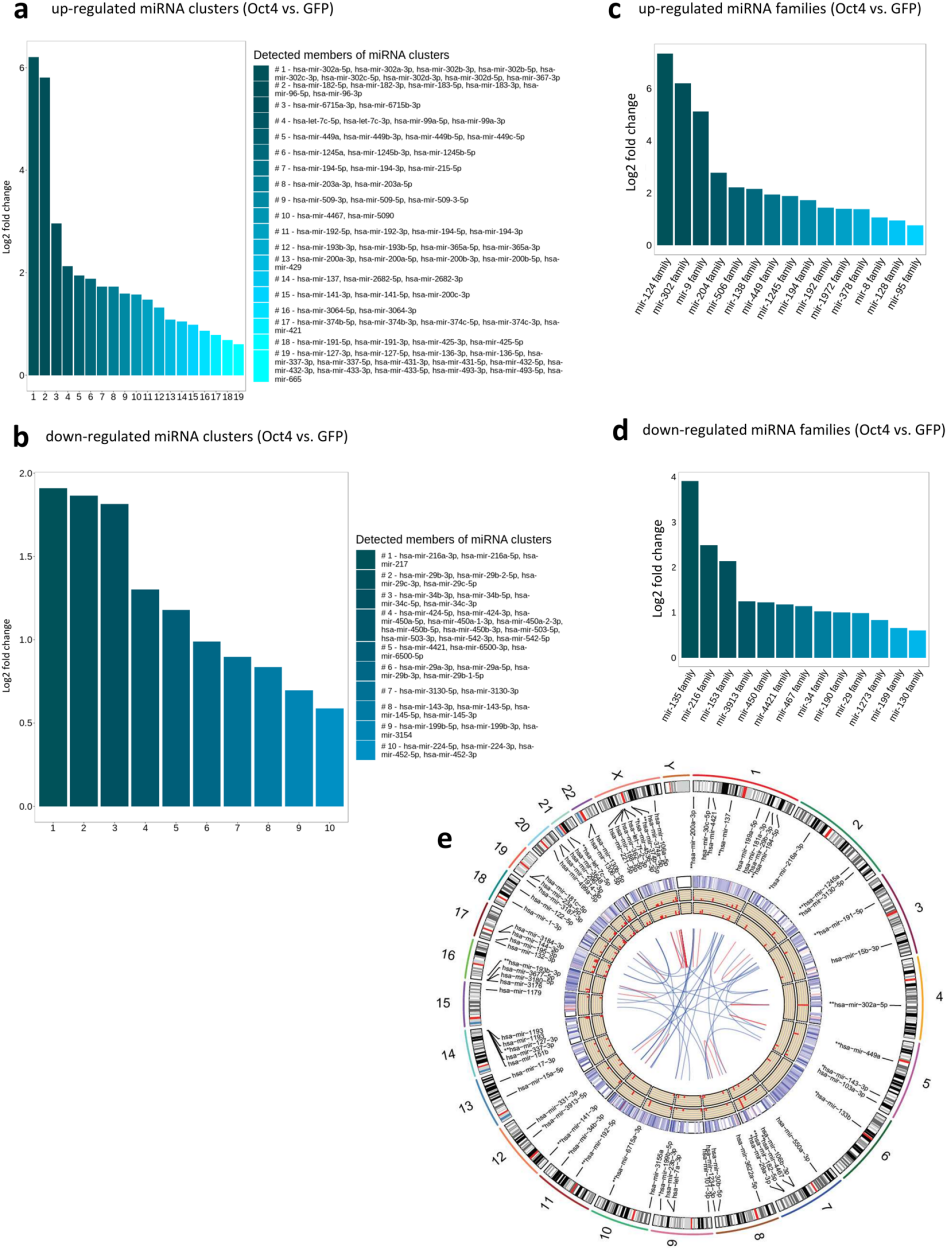
miRNA clusters and families analysis. **(a)** Up-regulated and **(b)** down-regulated miRNA clusters in Oct4+ hDFs. **(c)** Up-regulated and **(d)** down-regulated miRNA families in Oct4+ hDFs. **(e)** The Circos plot showing genomic locations and expression of miRNA clusters in Oct4+ and GFP+ hDFs. The outer circle represents chromosome ideogram as the reference for the genomic localization of individual miRNAs and clusters. Names of miRNA clusters are represented by name of the first miRNA molecule in a particular cluster. Clusters significantly up-regulated in Oct4+ hDFs are marked with two asterisks (**), clusters significantly down-regulated in Oct4+ hDFs are marked with one asterisk (*), (p < 0.05, Fold change(log2) > 1.5). The expression level of individual miRNAs (heatmap), miRNA clusters in Oct4+, and miRNA clusters in GFP+ hDFs are shown in the external, middle, and inner circles, respectively. The lines in the centre area represent interconnections between miRNA clusters and families. Red lines represent miRNA clusters that are also families.

It becomes increasingly clear that a single unique miRNA can be associated with specific cell functions. However, frequently miRNAs act in concert, being transcribed as clusters and/or acting as miRNA families, targeting the same target(s) or pathways(s). Therefore, better understanding of the function of the miRNAome is given rather by analysing the whole miRNA groups than individual miRNA molecules. We performed clustering of all expressed miRNAs based on their genomic cluster position (miRNA clusters, arbitrarily defined in miRBase as miRNAs located within 10,000 bases) and seed sequence similarities (miRNA families). We found 19 clusters and 15 miRNA families to be up-regulated and 10 clusters and 13 families to be down-regulated in hDFs over-expressing Oct4 when compared to GFP controls (p < 0.05, fold change > 1.5) (Fig. 5a–d).

In many scenarios, the genomic locations of differentially expressed miRNAs are not random, but they are clustered into specific loci of the genome. It is possible that these loci share the same regulatory pathways, however little is known about them. We visualised these loci into Circos plot, which shows a disperse distribution of the loci across the genome, with chromosome 1 being the most enriched with detected differentially expressed miRNA clusters followed by chromosomes 2 and 7 (Fig. 5e). Interestingly, many differentially expressed miRNA clusters are also miRNA families (red lines in the centre area), for example: mir-302, mir-182, mir-192, and mir-34.

**Figure 6:**
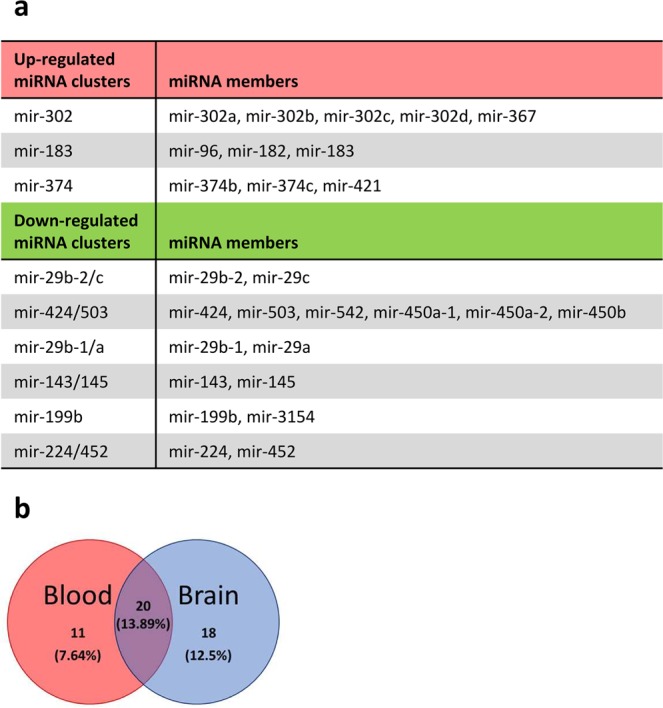
(**a**) miRNA clusters found to be significantly down- or up-regulated in Oct4+ hDFs as well as in human pluripotent stem cells. **(b)** Venn diagram shows number of miRNAs that are up-regulated in Oct4+ hDFs and also found to be highly-expressed in human blood and/or brain tissues (according to miRmine database).

It has been shown that mir-302 and mir-182 clusters are highly-expressed and mir-143/145 cluster is down-regulated in pluripotent stem cells^21,25,32,33^. To prospectively determine whether differentially expressed miRNA clusters share expression pattern with pluripotent stem cells, we compared these differentially expressed miRNA clusters in Oct4+ hDFs with miRNA expression in iPSCs^25^ (GEO: GSE68672). We found that three clusters (mir-302, mir-183, and mir-374, containing 21 mature miRNAs) that are expressed in pluripotent stem cells are also highly expressed in Oct4+ hDFs and six clusters (mir-29b-2/c, mir-424/503, mir-29b-1/a, mir-143/145, mir-199b, and mir-224/452, containing 30 mature miRNAs) were down-regulated in both Oct4+ hDFs and pluripotent stem cells when compared to GFP+ hDFs, indicating that Oct4+ hDFs share miRNA cluster expression similarities with pluripotent stem cells (Fig. 6a).

As Oct4 over-expression in fibroblasts promotes cell fate switch into the neural and hematopoietic lineages, we sought to compare the list of up-regulated miRNAs with miRNAs expressed in neural and hematopoietic lineages. We used miRmine database^34^ that provides a global view on human tissue miRNA expression profiles data. We found that 12.5% (18 miRNAs) of upregulated miRNAs were also found to be expressed in brain, 7.64% (11 miRNAs) are found to be expressed in blood, and 13.89% (20 miRNAs) were expressed in both brain and in blood (Fig. 6b).

### Over-expression of mir-302 cluster in hDFs alters cell morphology and down-regulates mesenchymal markers N-cadherin and β-catenin

**Figure 7:**
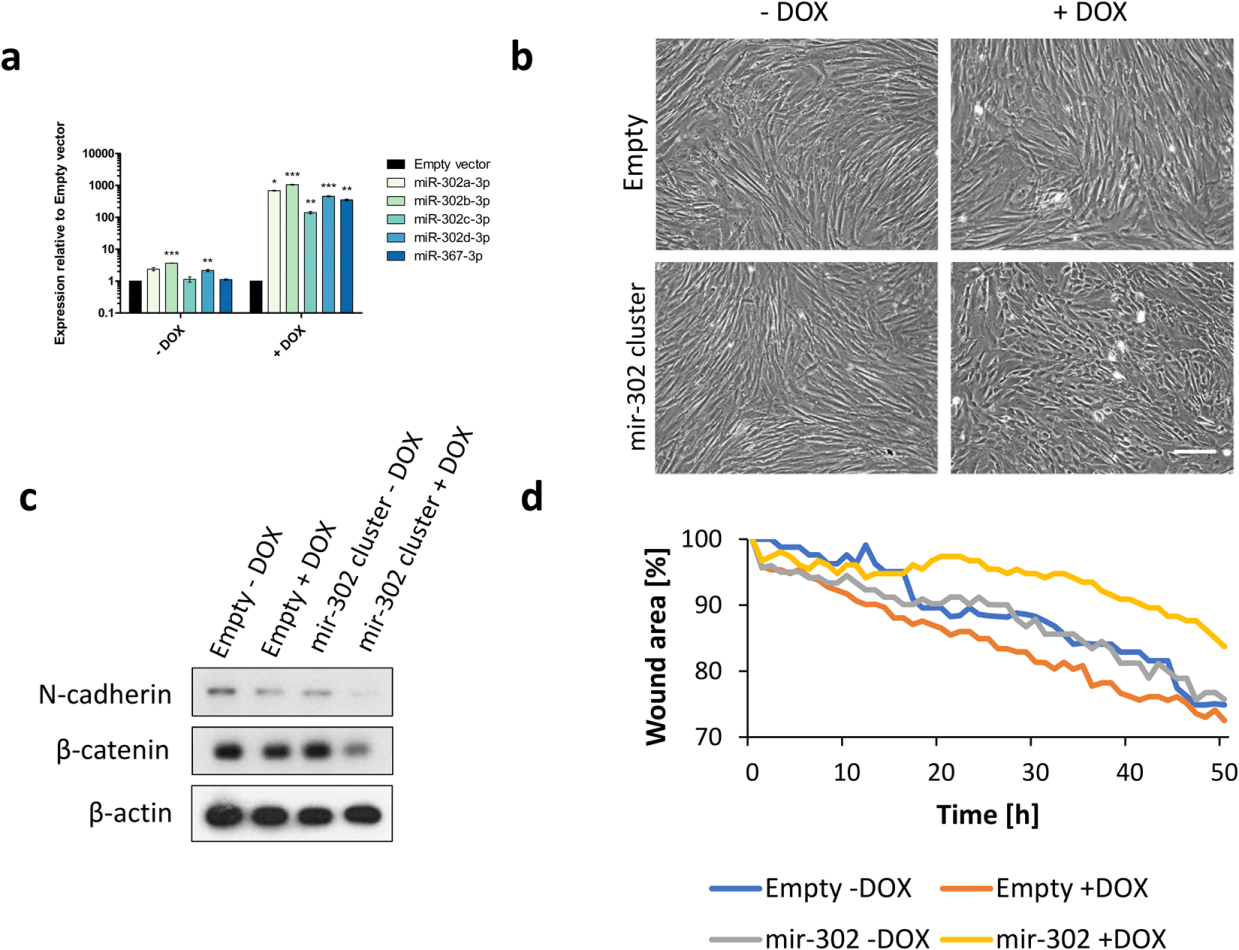
**(a)** Expression of individual members of mir-302 cluster upon Doxycycline (DOX)-inducible mir-302 cluster over-expression in hDFs, as determined by RT-qPCR. **(b)** Morphology of hDFs upon over-expression of mir-302 cluster, as determined by light microscopy. Scale bar = 100 μm. **(c)** Western blot analysis of N-cadherin and β-catenin expression in hDFs over-expressing mir-302 cluster. β-actin was used as a loading control. Uncropped western blot images are shown in Supplementary Fig. 2b. **(d)** Analysis of cell migration of hDFs over-expressing mir-302 cluster, as determined by scratch-wound assay. The graph shows cell-free area during time upon making a straight scratch on tissue culture plate.

Mir-302 cluster is the most up-regulated miRNA cluster in Oct4+ hDFs (Figs 3a and 5a) and it is classically viewed as a pluripotency-associated miRNA cluster that promotes reprogramming to iPSCs and it is also capable of altering cell fate^35^. We over-expressed mir-302 cluster in hDFs using Doxycycline-inducible vector (Fig. 7a). Similarly to Oct4 over-expression, cells changed their morphology upon mir-302 over-expression with transition of long-spindled fibroblast morphology to short-spindled cell shape that was associated with altered migration capacity of cells, and down-regulated mesenchymal markers N-cadherin and β-catenin (Fig. 7b–d).

## Discussion

Reprogramming of somatic cells to iPSCs is of great research importance and high hopes have been pinned on the potential application of iPSCs in regenerative medicine. However, time requirements and high costs of the reprogramming process, as well as possible tumorigenicity, immunogenicity and increased genomic instability of the resulting cells represent the major limitations towards practical application of iPSCs^36,37^. Therefore, an alternative reprogramming approach involving cell plasticity induction has been proposed.

Cell fate conversion using Oct4-mediated cell plasticity induction is a complex process regulated by a large number of gene networks and signalling pathways^4,5^, potentially containing also miRNAs. MiRNAs, a family of small non-coding RNAs that regulate gene expression, represent the key regulators of virtually all processes in cells and it is well documented that manipulations with single or multiple miRNAs can influence altering cell fate (reviewed in^21^). Still, the role of miRNAs in this process is not fully understood.

Here we show that Oct4 expression in hDFs down-regulates mesenchymal-related, up-regulates epithelial-related genes, and alters cell migration, suggesting that MET might play a role in this process. MET is crucial for early stage of the reprogramming process towards the generation of iPSCs^38,39^, however it is not clear whether MET is crucial for Oct4-mediated plasticity. In the first study describing the Oct4-induced plastic cells, MET has not been observed presumably due to different culture media, containing bFGF and IGF2, used in this study^5^. However, the authors observed profound changes in Oct4+ fibroblasts to acquire cuboidal shape that should represent a predictor for subsequent cell conversion competency, thus the involvement of epithelial/mesenchymal-associated genes in this process remains to be determined. It has been also demonstrated that Oct4-induced plasticity is characterized by mixed expression of lineage-specific genes^5^. We have not found any significant up-regulation of selected neuro- or cardio-associated genes (Fig. S4). However, we did not use media containing linage-inducing growth factors in our experimental setup, thus the mixed expression of lineage-specific genes may require the presence of these growth factors.

Our small RNA-Seq analysis revealed that Oct4+ hDFs possess distinct miRNA expression profile when compared to controls. We found more than 200 differentially expressed miRNAs upon Oct4 expression in hDFs. Interestingly, the data shows similarities with our previous results from miRNA microarray analysis comparing hDFs and iPSCs^25^. Among the top significantly down-regulated miRNAs clusters in hDFs over-expressing Oct4 are mir-424/503 and mir-34 clusters, whose members (e.g. mir-424, mir-503, mir-542, mir-34c), are found to be down-regulated in iPSCs as well^21,25^. We also found mir-143/145 cluster to be down-regulated upon Oct4 over-expression corroborating our previous findings that miR-145-5p is highly expressed in fibroblasts and inhibition of this miRNA in fibroblasts induces MET and facilitates reprogramming into iPSCs^25^. Surprisingly, we found embryonic-specific mir-302 and mir-183 clusters significantly up-regulated upon Oct4-induced plasticity in hDFs. Mir-302 cluster (comprised of mir-302a, mir-302b, mir-302c, mir-302d and mir-367) is referred to as embryonic stem cell-specific miRNA cluster that maintains pluripotency and ensures short G1 phase, therefore preserving unique properties of undifferentiated pluripotent cells^40^. It is also of note, that mir-302 cluster (alone or in combination with small molecules) has been shown to reprogram differentiated cells into iPSCs^41^. Interestingly, hDFs inducibly over-expressing mir-302 cluster show similar features as Oct4+ hDFs shortly upon induction – cuboidal shape, changes in the levels of mesenchymal markers and altered cell migration.

One could argue that mir-302 cluster up-regulation in Oct4+ hDFs is not so surprising, as Oct4 directly activates its transcription in pluripotent stem cells^40,42^. However, in OSKM reprogramming mir-302 cluster is induced only late in the process, moreover in embryonic stem cells its expression requires Oct4 in orchestration with Sox2 in order to maintain pluripotent state^21,25,40^. Thus, mir-302 cluster up-regulation shortly upon Oct4 over-expression is rather unexpected, indicating a unique molecular profile of Oct4+ hDFs. Similarly, members of mir-183 cluster (mir-96, mir-182 and mir-183) are expressed at high levels in pluripotent stem cells and are gradually expressed only during the late stage of OSKM reprogramming^43,44^. On the contrary, we did not detect significantly elevated levels of other pluripotent stem cells-enriched miRNAs, such as mir-205, mir-17-92 cluster, mir-371-373 cluster^21,33^, suggesting that these miRNAs are not crucial for Oct4-mediated cell fate alterations and underscoring the unique miRNA expression profile of Oct4+ hDFs.

As we have detected some differentially expressed miRNA clusters to share the expression pattern with pluripotent stem cells, one could expect that Oct4-mediated cell fate switch requires a brief transition through pluripotent state. It has been demonstrated that brief exposure to OSKM factors and subsequent cell fate switch requires this transition^8,9^, however global gene expression profiling of Oct4+ fibroblasts did not reveal any up-regulation of genes associated with pluripotency or genes typically expressed during early or late stage of iPSCs reprogramming process^5^. This is in concordance with miRNA expression profile of Oct4+ hDFs indicating mixed miRNA expression, however biased towards embryonic-like pattern. Together these finding indicate that forced Oct4 expression in fibroblasts leads to unique cell plastic state associated with gene and miRNA expression profile that is distinct from cells undergoing reprogramming towards iPSCs.

## Methods

### Human dermal fibroblast cells culture

All experiments were performed using three independent cell lines of human neonatal dermal fibroblasts NHDF-Neo (Lonza), here referred to as hDF1, hDF2, and hDF3. Fibroblast cells were cultured in Knockout Dulbecco’s modified Eagle’s medium (DMEM), (Invitrogen, Life Technologies Ltd.) containing 10% foetal bovine serum (FBS), (PAA), 2 mM L-glutamine (Invitrogen, Life Technologies Ltd.), 1 × MEM non-essential amino acid solution, 1 × penicillin/streptomycin (PAA) and 10 μM β-mercaptoethanol (Sigma-Aldrich). The cells were incubated at 37 °C/5% CO_2_.

### Preparation of pCW57-GFP-mir-302cluster vector for inducible mir-302 cluster over-expression

To over-express the members of the mir-302 cluster, we cloned mir-302 cluster sequence into Doxycycline inducible vector. Mir-302 cluster sequence was PCR-amplified from genomic DNA using primers containing AgeI/BsrGI restriction sites (forward primer: ACACCGGTGAAGTTGTATGTTGGGTGGGCT, reverse primer: CGTGTACACGTTATTTAACAATCCATCACCATTGCTAAAGT) and cloned into pJET1.2 using CloneJET PCR Cloning Kit (Thermo Fisher Scientific). pJET1.2 was AgeI/BsrGI digested and generated fragment was inserted into pCW57-GFP-2A-MCS vector using the same restriction sites. pCW57-GFP-2A-MCS was a gift from Adam Karpf (Addgene plasmid #71783; http://n2t.net/addgene:71783; RRID:Addgene_71783^45^. The pCW57-GFP-miR-302cluster vector is available at Addgene plasmid repository (www.addgene.org), Addgene plasmid #132549.

### Lentiviral particles production and infection

Lentiviral particles were generated, as described elsewhere^46^. Briefly, HEK293T cells were transfected with vectors for Oct4 over-expression pSin-EF2-Oct4-Pur (a gift from James Thomson), (Addgene #16579)^47^, or GFP (for control experiments) PL-SIN-EF1α-EGFP (a gift from James Ellis) (Addgene #21320)^48^, or pCW57-GFP-miR-302cluster (for mir-302 over-expression experiments), or pCW57-GFP-2A-MCS (for control experiments) together with 2^nd^ generation of lentiviral production plasmids psPAX2 (Addgene #12260) and pMD2.G (Addgene #12259) kindly provided by Didier Trono. Supernatant from “lentivirus-production” culture medium (OptiMEM [Invitrogen, Life Technologies Ltd.] containing 1% FBS, 1% MEM non-essential amino acid solution, 1% penicillin/streptomycin, 4 mM caffeine and 1 mM sodium butyrate [Sigma-Aldrich]) was collected every 12 hours for a total of 36 hours. Virus supernatant was centrifuged (4.500 × g, 10 minutes, room temperature), filtered through a 0.45 μm low protein-binding filter and concentrated using Vivaspin 20 columns with a 100 kDa molecular weight cut-off (VS2042, Sartorius). The concentrated virus supernatant was mixed with Polybrene (Sigma-Aldrich) at final concentration of 5 μg/ml and applied to fibroblast cells overnight. The next day, the culture medium containing viral particles was replaced with a fresh medium. For Puromycin selection, the culture medium was supplemented with 0.7 μg/ml Puromycin. For induction of mir-302 expression, the culture medium was supplemented with 1 μg/ml Doxycycline.

### RNA isolation and RT-qPCR

Total RNA from fibroblasts was isolated using RNA Blue Reagent (Top-Bio) as described previously^32^, and RNA integrity was analysed (RIN > 8). The isolated RNA was reverse transcribed (16 °C, 30 minutes; 42 °C, 30 minutes; 85 °C, 5 minutes) using TaqMan® MicroRNA Reverse Transcription Kit and specific primers for RNU6B, miR-96-5p, miR-182-5p, miR-183-5p, miR-302a-3p, miR-302b-3p, miR-302c-3p, miR-302d-3p, miR-34c-5p, miR-193a-5p, miR-424-5p, miR-503-5p, miR-143-3p, miR-145-5p, and miR-9-5p (Applied Biosystems). Reverse transcription products were then amplified by quantitative PCR (95 °C, 5 minutes; 95 °C, 15 seconds, 60 °C, 60 seconds, 40 cycles) (LightCycler® 480, Roche) using TaqMan Universal PCR Master Mix and specific probes for miRNAs. Relative microRNA expression was determined using ΔΔC_t_ method and normalized to endogenous control RNU6B.

For gene expression assays, the isolated RNA was reverse transcribed using Transcriptor First Strand cDNA Synthesis Kit with Anchored-oligo(dT)_18_ Primer (Roche). Reverse transcription products were then amplified by quantitative PCR (LightCycler® 480, Roche) using LightCycler® 480 SYBR Green I Master. The primers used in this study are listed in the Fig. S7. Relative gene expression was determined using ΔΔC_t_ method and normalized to endogenous control GAPDH.

### Western blot analysis

Western blot analysis was performed as described previously^46^. Briefly, cells were washed three times with PBS (pH 7.4) and lysed in buffer containing 50 mM Tris-HCl (pH 6.8), 10% glycerol and 1% sodium dodecyl sulfate (SDS). The lysates were homogenized by sonication and protein concentrations were determined using DC Protein Assay (Bio-Rad). The lysates were then supplemented with 0.01% bromophenol blue and 1% β-mercaptoethanol and denatured at 100 °C for 5 minutes. Prepared samples were separated by SDS-polyacrylamide gel electrophoresis and transferred onto polyvinylidene fluoride (PVDF) membrane (Merck Millipore). PVDF membrane was blocked in 5% skimmed milk in Tris-buffered saline containing Tween for 1 hour and incubated with primary antibodies overnight at 4 °C. The following antibodies were used: ZO-1 (#8193), Slug (#9585), N-cadherin (#13116), Snail (#3879), Vimentin (#5741), β-actin (#4970), β-catenin (#8480) all purchased from Cell Signaling Technology, Oct3/4 (sc-5279, Santa Cruz Biotechnology) and α-tubulin (11-250-C100, Exbio).

### NGS and library preparation

NEBNext Multiplex Small RNA Library Prep Set for Illumina (Set1, 2; New England Biolabs; Ipswich, MA, USA) was used to prepare libraries for further sequencing according to the manufacturer’s instructions as described previously^49^. Briefly, 800 ng of total RNA were used to create size selected small RNA library. The sequencing (single end) was performed with 2.0 pM library using the NextSeq® 500/550 High Output Kit v2 (75 cycles; Illumina; San Diego, CA, USA).

### NGS data analysis

The data were processed as described previously^49^. Briefly, the quality of the raw sequencing data was assessed using FastQC (v0.11.5)^50^, Minion and Swan (Kraken package, v15-065)^51^ were used to scan and identify adaptor sequences which were subsequently removed by Cutadapt (v1.12)^52^. Only adapter-containing reads were kept for further processing. The adapter-trimmed reads were further processed using the following steps: (1) Removal of very low-quality read ends (Phred < 5); (2) Keeping only reads with Phred score of 10 over at least 85% of the length; (3) Only reads within 16–28 bp were kept as potential microRNA reads. FASTX-Toolkit (v0.0.14)^53^ was used for the quality filtering, the rest of the steps were performed by Cutadapt (v1.12)^52^ and bash scripting. FastQ Screen (v0.10.0)^54^ and Bowtie (v1.1.2)^55^ were used to evaluate overall mapping of the preprocessed reads to the human reference genome (hg38)^56^ and to determine possible contamination by rRNA and tRNA molecules. An additional quality check was performed by Sequence Imp (v15-075)^51^. The raw microRNA expression levels were quantified by Chimira (v1.0)^57^. All data analysis and visualizations were conducted using R 3.4.4^58^ with installed Bioconductor project^59^. The following R packages were used to analyse and visualize data: DESeq2^60^, ggplot2^61^, pheatmap^62^, RColorBrewer^63^, plotly^64^, Mirbase.db^65^, and RCircos^66^ packages were used to cluster and visualize miRNAs into families and clusters.

### Scratch-wound assay

Cells were plated into 12-well plate (150,000 cells/well), cultured for additional three days and then scratched using a 100 µl pipette tip. Plates were photographed immediately after scratching (5 positions for each condition) and then every 1 hour for total of 50 hours using time-laps Cell R imaging station (Olympus). Cell-free area was measured using ImageJ (https://imagej.nih.gov/ij/) software.

## Supplementary information

Supplementary Information

## Data Availability

The datasets generated during and/or analysed during the current study are available in the GEO database repository (https://www.ncbi.nlm.nih.gov/geo/), accession number: GSE126284

## References

[1] Takahashi K (2007). Induction of pluripotent stem cells from adult human fibroblasts by defined factors. Cell.

[2] Takahashi K, Yamanaka S (2006). Induction of pluripotent stem cells from mouse embryonic and adult fibroblast cultures by defined factors. Cell.

[3] Graf T (2011). Historical origins of transdifferentiation and reprogramming. Cell Stem Cell.

[4] Mitchell RR (2014). Activation of Neural Cell Fate Programs Toward Direct Conversion of Adult Human Fibroblasts into Tri-Potent Neural Progenitors Using OCT-4. Stem Cells Dev..

[5] Mitchell R (2014). Molecular Evidence for OCT4-Induced Plasticity in Adult Human Fibroblasts Required for Direct Cell Fate Conversion to Lineage Specific Progenitors. STEM CELLS.

[6] Szabo E (2010). Direct conversion of human fibroblasts to multilineage blood progenitors. Nature.

[7] Kelaini S, Cochrane A, Margariti A (2014). Direct reprogramming of adult cells: avoiding the pluripotent state. Stem Cells Cloning Adv. Appl..

[8] Bar-Nur O (2015). Lineage conversion induced by pluripotency factors involves transient passage through an iPSC stage. Nat. Biotechnol..

[9] Maza I (2015). Transient acquisition of pluripotency during somatic cell transdifferentiation with iPSC reprogramming factors. Nat. Biotechnol..

[10] Margariti A (2012). Direct reprogramming of fibroblasts into endothelial cells capable of angiogenesis and reendothelialization in tissue-engineered vessels. Proc. Natl. Acad. Sci..

[11] Prasad A (2016). A review of induced pluripotent stem cell, direct conversion by trans-differentiation, direct reprogramming and oligodendrocyte differentiation. Regen. Med..

[12] Karamariti E (2013). Smooth muscle cells differentiated from reprogrammed embryonic lung fibroblasts through DKK3 signaling are potent for tissue engineering of vascular grafts. Circ. Res..

[13] Doffou M (2018). Oct4 Is Crucial for Transdifferentiation of Hepatocytes to Biliary Epithelial Cells in an *In Vitro* Organoid Culture Model. Gene Expr..

[14] Kim J (2011). Direct reprogramming of mouse fibroblasts to neural progenitors. Proc. Natl. Acad. Sci..

[15] Efe JA (2011). Conversion of mouse fibroblasts into cardiomyocytes using a direct reprogramming strategy. Nat. Cell Biol..

[16] Li X (2016). Reversine Increases the Plasticity of Long-Term Cryopreserved Fibroblasts to Multipotent Progenitor Cells through Activation of Oct4. Int. J. Biol. Sci..

[17] Radzisheuskaya A, Silva JCR (2014). Do all roads lead to Oct4? The emerging concepts of induced pluripotency. Trends Cell Biol..

[18] Salci KR (2015). Acquisition of pluripotency through continued environmental influence on OCT4-induced plastic human fibroblasts. Stem Cell Res..

[19] Zhu S (2010). Reprogramming of Human Primary Somatic Cells by OCT4 and Chemical Compounds. Cell Stem Cell.

[20] Wang Y (2011). Reprogramming of mouse and human somatic cells by high-performance engineered factors. EMBO Rep..

[21] Moradi, S., Asgari, S. & Baharvand, H. Concise Review: Harmonies Played by MicroRNAs in Cell Fate Reprogramming. *STEM CELLS* **32**, 3–15 (2014). 10.1002/stem.1576 24155164

[22] Huntzinger E, Izaurralde E (2011). Gene silencing by microRNAs: contributions of translational repression and mRNA decay. Nat. Rev. Genet..

[23] Onder TT, Daley GQ (2011). microRNAs become macro players in somatic cell reprogramming. Genome Med..

[24] Parchem RJ (2014). Two miRNA clusters reveal alternative paths in late-stage reprogramming. Cell Stem Cell.

[25] Barta T (2016). Brief Report: Inhibition of miR-145 Enhances Reprogramming of Human Dermal Fibroblasts to Induced Pluripotent Stem Cells. Stem Cells Dayt. Ohio.

[26] Ying S-Y, Fang W, Lin S-L (2018). The miR-302-Mediated Induction of Pluripotent Stem Cells (iPSC): Multiple Synergistic Reprogramming Mechanisms. Methods Mol. Biol. Clifton NJ.

[27] Lin S-L (2008). Mir-302 reprograms human skin cancer cells into a pluripotent ES-cell-like state. RNA N. Y. N.

[28] Lin S-L (2011). Regulation of somatic cell reprogramming through inducible mir-302 expression. Nucleic Acids Res..

[29] Miyoshi N (2011). Reprogramming of mouse and human cells to pluripotency using mature microRNAs. Cell Stem Cell.

[30] Unternaehrer JJ (2014). The epithelial-mesenchymal transition factor SNAIL paradoxically enhances reprogramming. Stem Cell Rep..

[31] Kozomara A, Griffiths-Jones S (2014). miRBase: annotating high confidence microRNAs using deep sequencing data. Nucleic Acids Res..

[32] Dolezalova D (2012). MicroRNAs regulate p21(Waf1/Cip1) protein expression and the DNA damage response in human embryonic stem cells. Stem Cells Dayt. Ohio.

[33] Houbaviy HB, Murray MF, Sharp PA (2003). Embryonic stem cell-specific MicroRNAs. Dev. Cell.

[34] Panwar B, Omenn GS, Guan Y (2017). miRmine: a database of human miRNA expression profiles. Bioinforma. Oxf. Engl..

[35] Ghasemi-Kasman M, Zare L, Baharvand H, Javan M (2018). *In vivo* conversion of astrocytes to myelinating cells by miR-302/367 and valproate to enhance myelin repair. J. Tissue Eng. Regen. Med..

[36] Martins‐Taylor K, Xu R-H (2012). Concise Review: Genomic Stability of Human Induced Pluripotent Stem Cells. Stem Cells.

[37] Neofytou E, O’Brien CG, Couture LA, Wu JC (2015). Hurdles to clinical translation of human induced pluripotent stem cells. J. Clin. Invest..

[38] Li R (2010). A mesenchymal-to-epithelial transition initiates and is required for the nuclear reprogramming of mouse fibroblasts. Cell Stem Cell.

[39] Samavarchi-Tehrani P (2010). Functional genomics reveals a BMP-driven mesenchymal-to-epithelial transition in the initiation of somatic cell reprogramming. Cell Stem Cell.

[40] Card DAG (2008). Oct4/Sox2-regulated miR-302 targets cyclin D1 in human embryonic stem cells. Mol. Cell. Biol..

[41] Anokye-Danso F (2011). Highly efficient miRNA-mediated reprogramming of mouse and human somatic cells to pluripotency. Cell Stem Cell.

[42] Hu S (2013). MicroRNA-302 increases reprogramming efficiency via repression of NR2F2. Stem Cells Dayt. Ohio.

[43] Dambal S, Shah M, Mihelich B, Nonn L (2015). The microRNA-183 cluster: the family that plays together stays together. Nucleic Acids Res..

[44] Polo JM (2012). A Molecular Roadmap of Reprogramming Somatic Cells into iPS Cells. Cell.

[45] Barger, C. J., Branick, C., Chee, L. & Karpf, A. R. Pan-Cancer Analyses Reveal Genomic Features of FOXM1 Overexpression in Cancer. *Cancers* **11** (2019). 10.3390/cancers11020251 PMC6406812 30795624

[46] Barta T, Peskova L, Hampl A (2016). miRNAsong: a web-based tool for generation and testing of miRNA sponge constructs in silico. Sci. Rep..

[47] Yu J (2007). Induced pluripotent stem cell lines derived from human somatic cells. Science.

[48] Hotta A (2009). Isolation of human iPS cells using EOS lentiviral vectors to select for pluripotency. Nat. Methods.

[49] Cerna Katerina, Oppelt Jan, Chochola Vaclav, Musilova Katerina, Seda Vaclav, Pavlasova Gabriela, Radova Lenka, Arigoni Maddalena, Calogero Raffaele A., Benes Vladimir, Trbusek Martin, Brychtova Yvona, Doubek Michael, Mayer Jiri, Pospisilova Sarka, Mraz Marek (2018). MicroRNA miR-34a downregulates FOXP1 during DNA damage response to limit BCR signalling in chronic lymphocytic leukaemia B cells. Leukemia.

[50] Babraham Bioinformatics. Available at: http://www.bioinformatics.babraham.ac.uk/index.html (Accessed: 22nd August 2018).

[51] Davis MPA, van Dongen S, Abreu-Goodger C, Bartonicek N, Enright AJ (2013). Kraken: a set of tools for quality control and analysis of high-throughput sequence data. Methods San Diego Calif.

[52] Martin M (2011). Cutadapt removes adapter sequences from high-throughput sequencing reads. EMBnet.journal.

[53] FASTX-Toolkit. Available at: http://hannonlab.cshl.edu/fastx_toolkit/index.html (Accessed: 22nd August 2018).

[54] Babraham Bioinformatics - FastQ Screen. Available at: https://www.bioinformatics.babraham.ac.uk/projects/fastq_screen/ (Accessed: 22nd August 2018).

[55] Langmead B, Trapnell C, Pop M, Salzberg SL (2009). Ultrafast and memory-efficient alignment of short DNA sequences to the human genome. Genome Biol..

[56] Speir ML (2016). The UCSC Genome Browser database: 2016 update. Nucleic Acids Res..

[57] Vitsios DM, Enright AJ (2015). Chimira: analysis of small RNA sequencing data and microRNA modifications. Bioinforma. Oxf. Engl..

[58] R: The R Project for Statistical Computing. Available at: https://www.r-project.org/ (Accessed: 22nd August 2018).

[59] Huber W (2015). Orchestrating high-throughput genomic analysis with Bioconductor. Nat. Methods.

[60] Love MI, Huber W, Anders S (2014). Moderated estimation of fold change and dispersion for RNA-seq data with DESeq2. Genome Biol..

[61] Wickham, H. *ggplot2: Elegant Graphics for Data Analysis*. (Springer-Verlag, 2009).

[62] Kolde, R. *pheatmap: Pretty Heatmaps* (2018).

[63] Neuwirth, E. *RColorBrewer: ColorBrewer Palettes* (2014).

[64] Sievert, C. *et al*. *plotly: Create Interactive Web Graphics via ‘plotly.js’* (2018).

[65] mirbase.db. *Bioconductor* Available at: http://bioconductor.org/packages/mirbase.db/ (Accessed: 22nd August 2018).

[66] Zhang H, Meltzer P, Davis S (2013). RCircos: an R package for Circos 2D track plots. BMC Bioinformatics.

