# Supplementary material for: Oct4-mediated reprogramming induces embryonic-like microRNA expression signatures in human fibroblasts
Source: Sci Rep. 2019 Oct 31;9:15759. doi: 10.1038/s41598-019-52294-3 (PMC6823439; doi:10.1038/s41598-019-52294-3)
Supplement: Supplementary file 1 — Supplementary Information [file 41598_2019_52294_MOESM1_ESM.pdf]

Supplementary information file

Manuscript: Oct4-mediated reprogramming induces embryonic-like microRNA expression signatures in human fibroblasts

Lucie Peskova[1], Katerina Cerna[2], Jan Oppelt[2,4], Marek Mraz[2,3], Tomas Barta[1*]

1) Department of Histology and Embryology, Faculty of Medicine, Masaryk University, Brno 625 00, Czech Republic
2) CEITEC-Central European Institute of Technology, Masaryk University, Brno 625 00, Czech Republic
3) Department of Internal Medicine, Hematology and Oncology, University Hospital Brno and Faculty of Medicine, Masaryk University, Brno, Czech Republic
4) National Centre for Biomolecular Research, Faculty of Science, Masaryk University, Brno 625 00, Czech Republic

Supplementary Figure 1 (Peskova et al.)

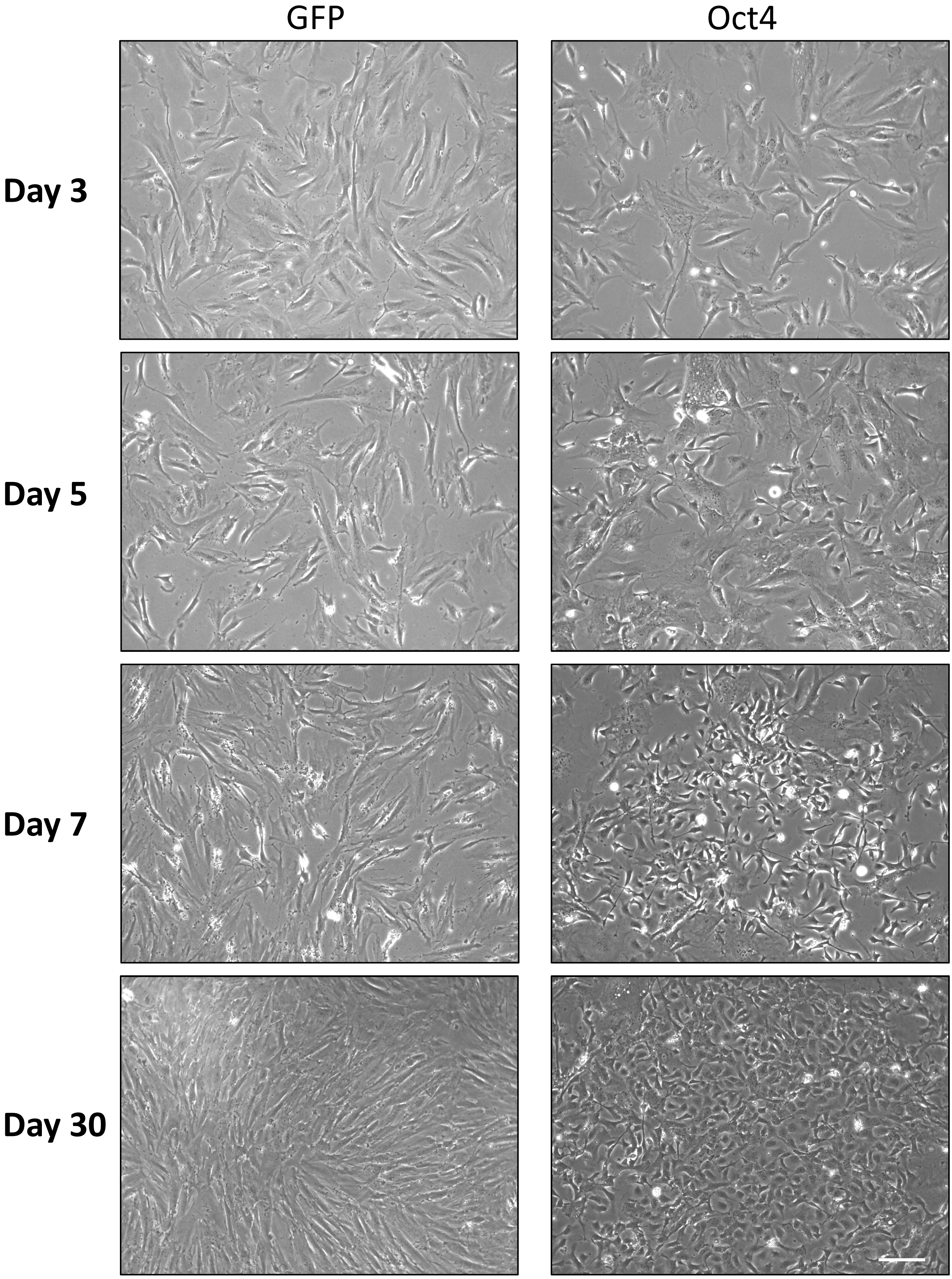

Supplementary Figure S1: Morphology of hDFs expressing GFP (control) or Oct4, as determined by bright field microscopy. Scale bar = 100 µm.

Supplementary Figure 2 (Peskova et al.)

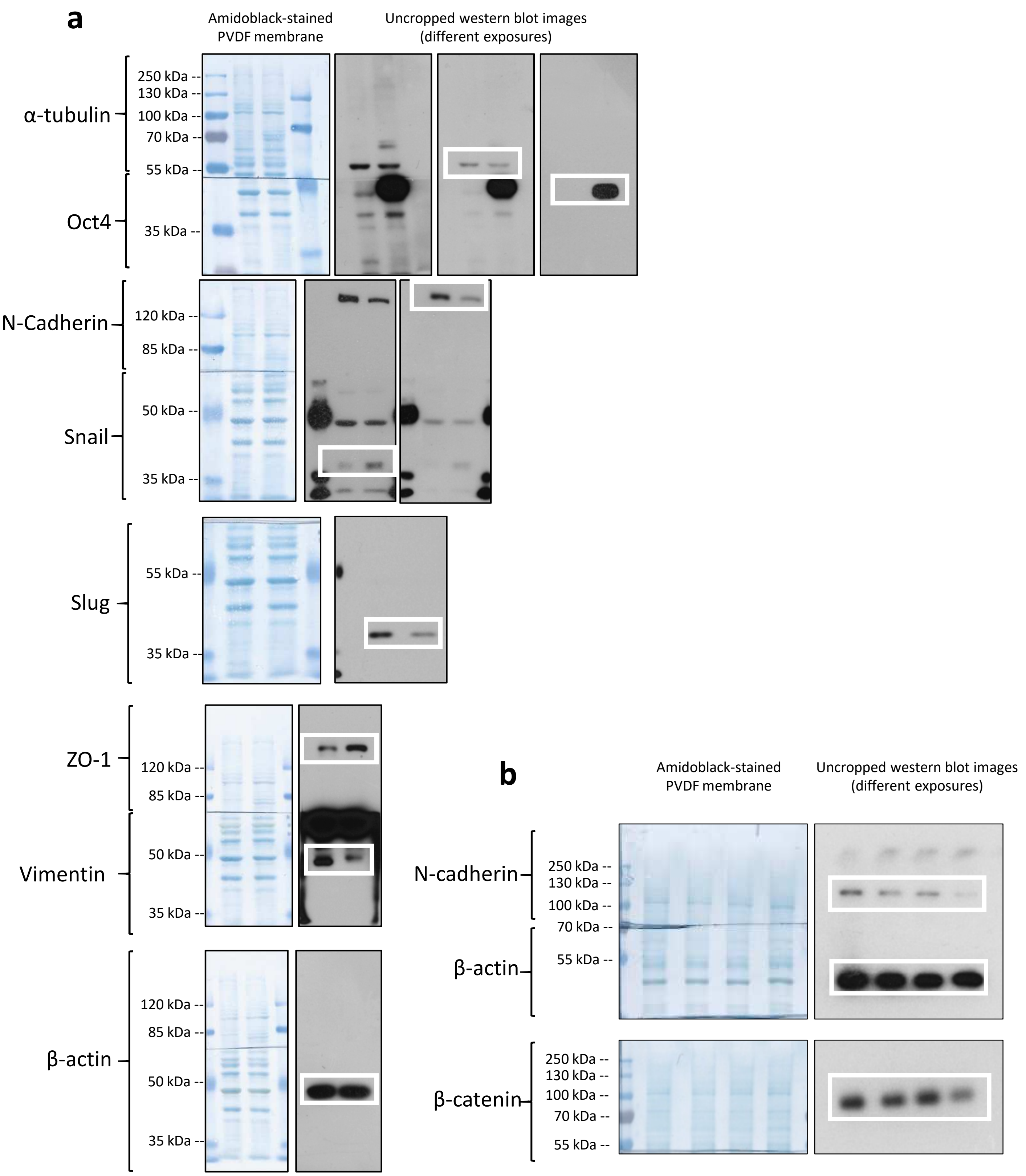

Supplementary Figure S2: (a) Western blot analysis of mesenchymal/epithelial markers and Oct4 expression in control GFP+ hDFs and Oct4+ hDFs 6 days post transduction. α-tubulin and β-actin were used as a loading control. Full-scan of western blot images presented in the Figure 1c with molecular weight marker indicated. Corresponding fragments of blot (white rectangles) used in the Figure 1c are indicated. **(b)** Western blot analysis of N-cadherin and β-catenin expression in hDFs over-expressing mir-302 cluster. β-actin was used as a loading control. Full-scan of western blot images presented in the Figure 7c with molecular weight marker indicated. Corresponding fragments of blot (white rectangles) used in the Figure 7c are indicated.

Supplementary Figure 3 (Peskova et al.)

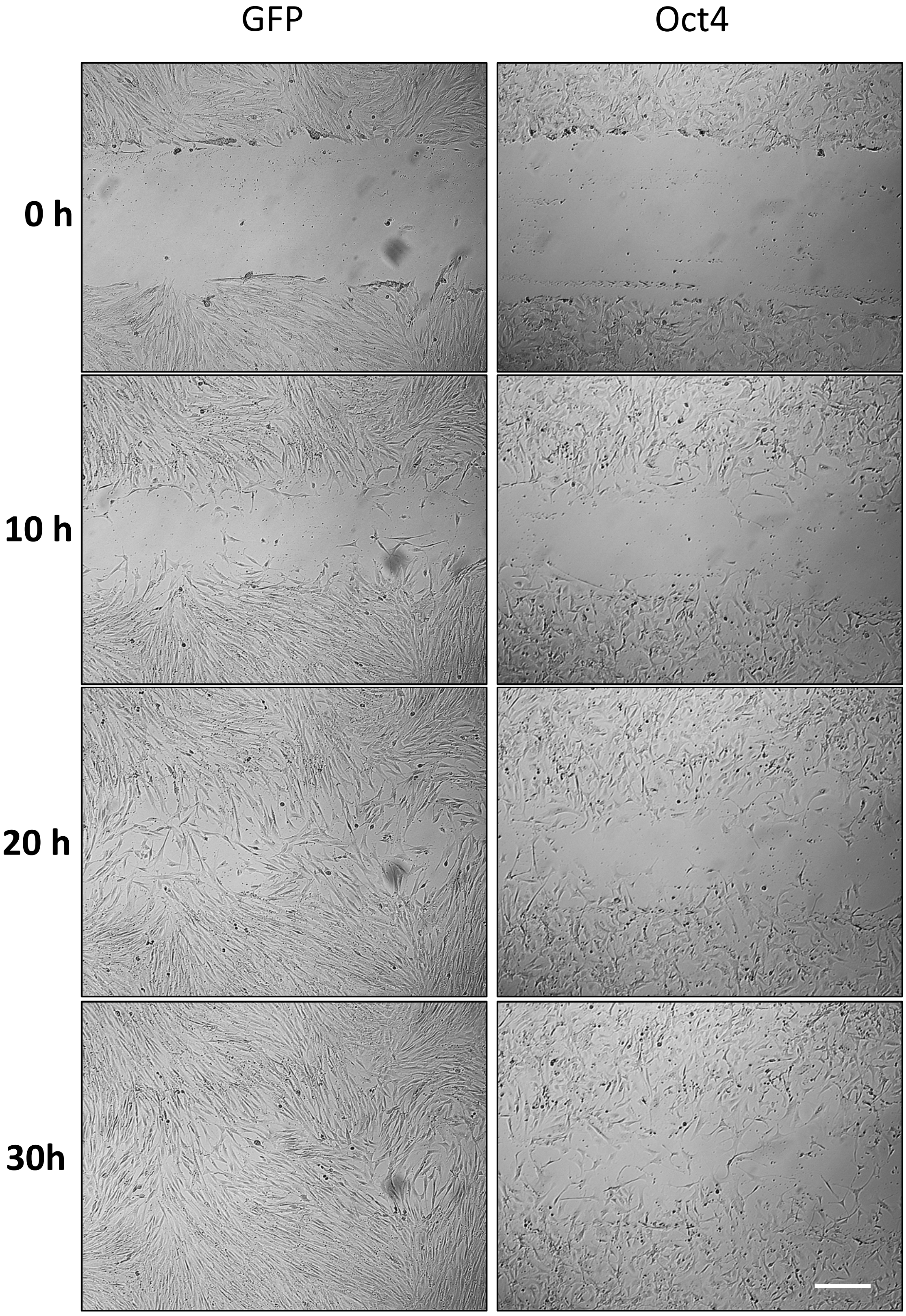

Supplementary Figure S3: Assessment of hDFs migration upon over-expression of GFP (control) or Oct4, as determined by time-lapse bright field microscopy. Scale bar = 100 µm.

Supplementary Figure 4 (Peskova et al.)

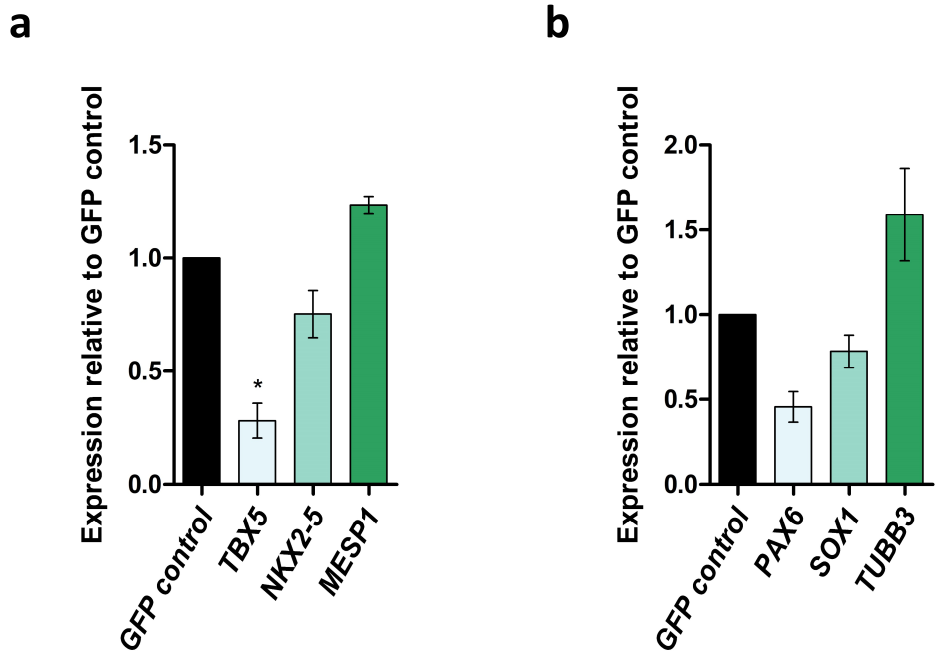

Supplementary Figure S4: Expression levels of a) *TBX5*, *NKX2-5*, *MESP1* and b) *PAX6, SOX1, TUBB3* upon Oct4 over-expression in hDFs, as determined using RT-qPCR.

Supplementary Figure 5 (Peskova et al.)

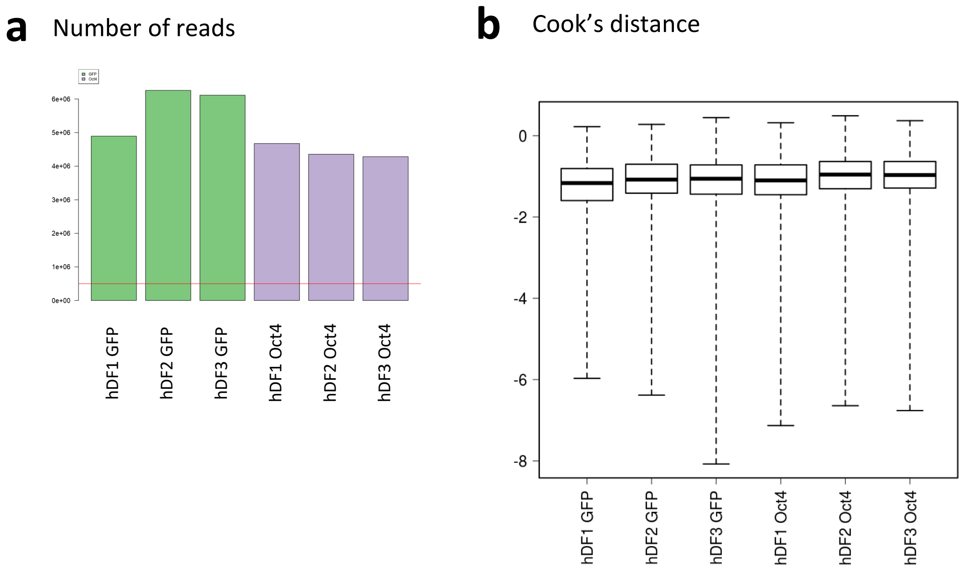

Supplementary Figure S5: NGS quality check. **(a)** Number of raw reads in individual samples. **(b)** Cook's distance of individual samples

Supplementary Figure 6 (Peskova et al.)

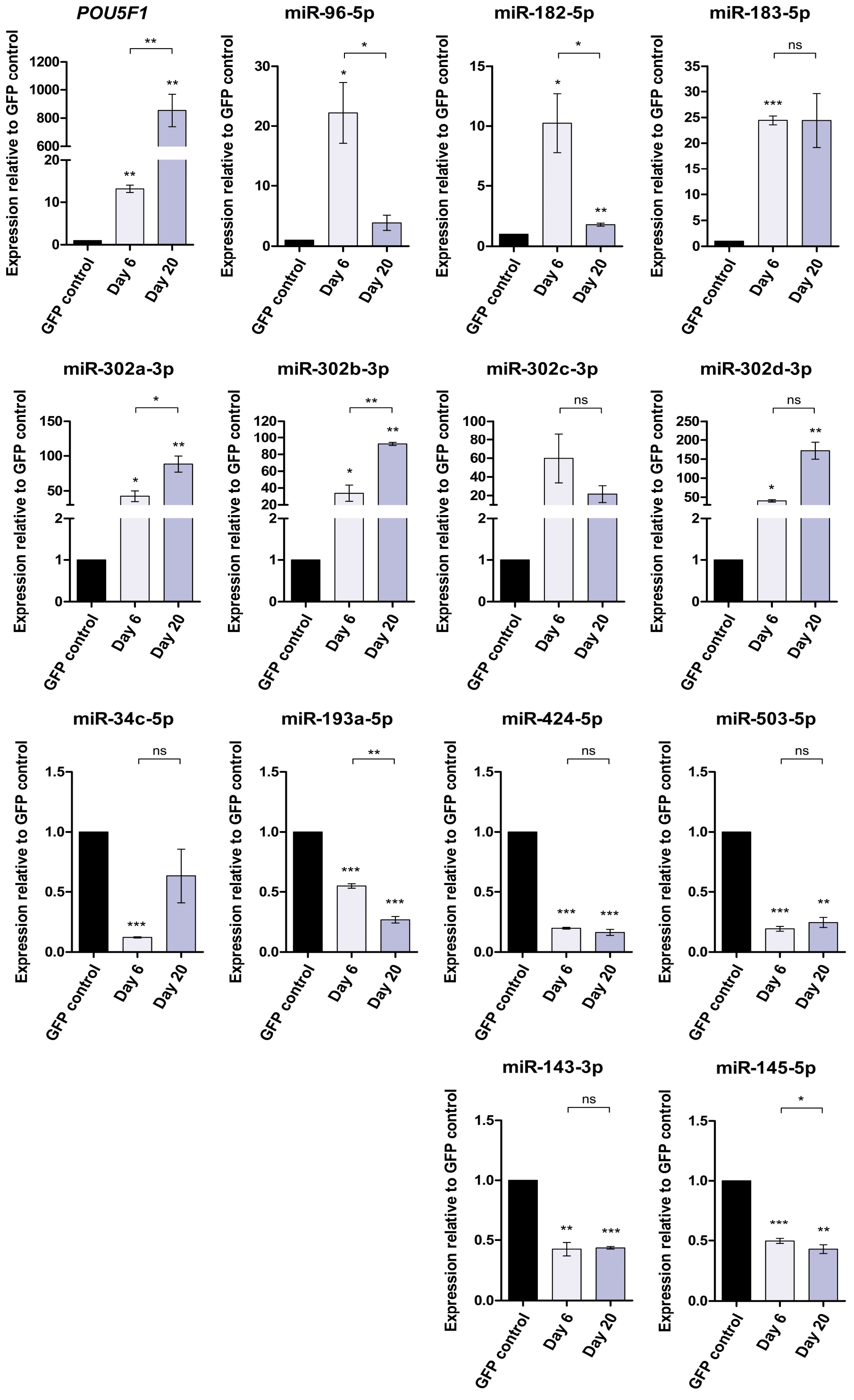

Supplementary Figure S6: Expression levels of *POU5F1* (Oct4) and selected miRNAs that are differentially expressed based on NGS data, as determined by RT-qPCR at day 6 and 20 upon transduction. The results are shown as expression relative to corresponding control GFP+ hDFs. Error bars represent ± SD.

Supplementary Figure 7 (Peskova et al.)

Primer	Sequence
GAPDH-Left	AGCCACATCGCTCAGACAC
GAPDH Right	GCCCAATACGACCAAATCC
SNAI2 Left	TGGTTGCTTCAAGGACACAT
SNAI2 Right	GCAAATGCTCTGTTGCAGTG
POU5F1 Left	GAAACCCACACTGCAGATCA
POU5F1 Right	CGGTTACAGAACCACACTCG
COL1A1 Left	CCCAAGGCTTCCAAGGTC
COL1A1 Right	GGACGACCAGGTTTTCCAG
CDH1 Left	TGGAGGAATTCTTGCTTTGC
CDH1 Right	CGCTCTCCTCCGAAGAAAC
EPCAM Left	CCATGTGCTGGTGTGTGAA
EPCAM Right	TGTGTTTTAGTTCAATGATGATCCA
CRB3 Left	GCACTGTTTTGCCTTCATCC
CRB3 Right	AGCAGTGATGGCTTCTGGAC
PAX6 Left	GCACACACACATTAACACACTTG
PAX6 Right	GGTGTGTGAGAGCAATTCTCAG
SOX1 Left	TCCCCCGCGTGAACTG
SOX1 Right	CAAGGCATTTTGCGTTCACA
TUBB3: QUANTITECT® PRIMER ASSAY HS_TUBB3_1_SG CAT.NO.: QT00083713	
NKX2-5 Left	CACCTCAACAGCTCCCTGA
NKX2-5 Right	CTAGGTCTCCGCAGGAGTGA
TBX5 Left	CCAGGAGCATAGCCAAATTTAC
TBX5 Right	AGGGCTTCTTATAGGGATGGTC
MESP1 Left	CACTTTGAGGCAAGCAGGA
MESP1 Right	GCCAACTGACACCAGTACAGTTTA

Supplementary Figure S7: The list of primers and their sequencies used in this study.

Supplementary Figure 8 (Peskova et al.)

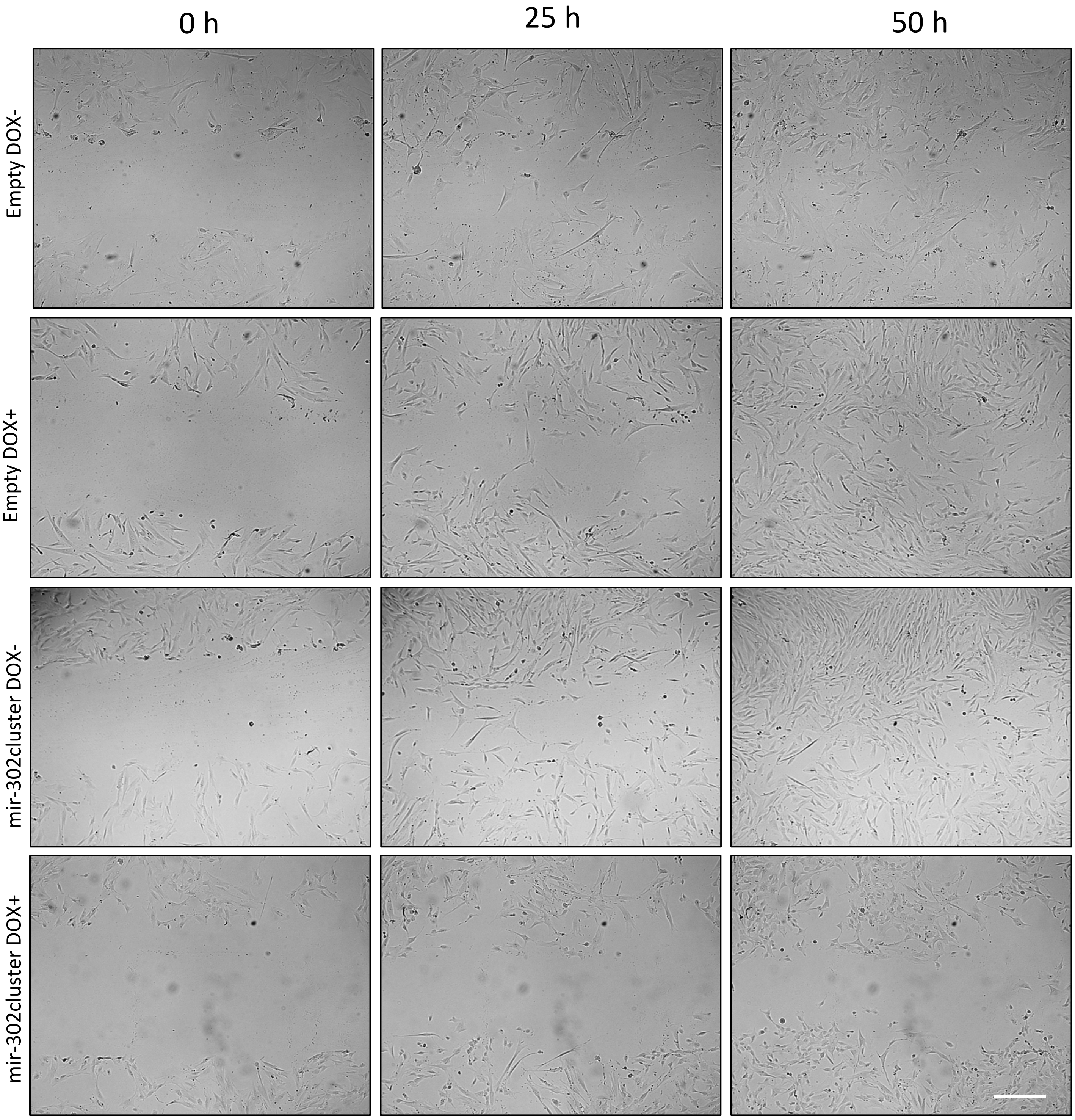

Supplementary Figure S8: Assessment of hDFs migration upon mir-302 cluster over-expression, as determined by time-lapse bright field microscopy. Cells expressing empty vector and the presence or absence of Doxycyline represent a control. Scale bar = 100 µm.
